# New species of *Uvariopsis* (Annonaceae) and *Laccosperma* (Arecaceae/Palmae) from Monts de Cristal, Gabon

**DOI:** 10.3897/phytokeys.68.9576

**Published:** 2016-08-02

**Authors:** Thomas L.P. Couvreur, Raoul Niangadouma

**Affiliations:** 1 Institut de Recherche pour le Développement, UMR-DIADE, BP 64501, F-34394 Montpellier cedex 5, France; 2 University of Yaoundé I, Higher Teacher’s Training College, Plant Systematic and Ecology Laboratory, P.O. Box 047, Yaoundé, Cameroon; 3 Naturalis Biodiversity Centre, Botany Section, Darwinweg 2, 2333 CR Leiden, The Netherlands; 4 National Herbarium of Gabon, R.D. 1135, Libreville, Gabon

**Keywords:** Annonaceae, lemon scent, national park, rattan, Gabon

## Abstract

Monts de Cristal National Park in northwest Gabon is one of the most species rich places in Central Africa. Here, we describe two new species, one in Annonaceae and one in palms. *Uvariopsis citrata* Couvreur & Niangadouma, **sp. nov.** is unique in the genus by emitting a strong lemon scent from the crushed leaves and young branches. *Laccosperma cristalensis* Couvreur & Niangadouma, **sp. nov.** is a rattan that lacks acanthophylls on the cirrus and has few pinnae. Complete descriptions, photographic illustrations, ecological information and preliminary IUCN conservation status are provided. For both species a data deficient (DD) status is proposed. These new species underline once again that the Monts de Cristal National Park is yet incompletely known botanically.

## Introduction

The Monts de Cristal National Park, in northwestern Gabon, is located less than 100 km from the capital Libreville. Monts de Cristal is one of the most plant species-rich areas in Central Africa (Sosef et al. 2006). Because of its prime locality, it is often visited by botanists and is one of the most densely botanically collected areas in Gabon (Wieringa and Sosef 2011). However, new species have been regularly described from the national park (e.g. Ječmenica et al. 2016; Stévart et al. 2014), including a new genus of Annonaceae, *Sirdavidia* (Couvreur et al. 2015) which was recently awarded the top 10 new species of 2016 (International Institute for Species Exploration 2016). Here, we describe two new species collected during a botanical trip to the Monts de Cristal National Park in June 2016, one Annonaceae and one rattan.

The genus *Uvariopsis* (Annonaceae) contains a total of 18 species restricted to Africa (Couvreur and Luke 2010; Gereau and Kenfack 2000; Kenfack et al. 2003). *Uvariopsis* is unique in African Annonaceae as most of its species have one whorl of 2 sepals and one whorl of 4 petals, in contrast to the typical Annonaceae pattern of 3 sepals, and 2 whorls of 3 petals. *Uvariopsis* belongs to the Monodoreae tribe and is recovered as sister to the genus *Monocyclanthus* (Chatrou et al. 2012; Couvreur et al. 2008).

*Laccosperma* is one of the four rattan genera found in Africa (Sunderland 2007). *Laccosperma* contains six species and belongs to the subtribe Ancistrophyllinae which also contains two other genera: *Oncocalamus* and *Eremospatha* (Sunderland 2007; Sunderland 2012). *Laccosperma* is distinguished from the latter two by the robust and rounded spines on its leaf sheath and stems coupled with hermaphroditic flowers (Sunderland 2012). *Laccosperma* was recently shown to be sister to *Eremospatha* (Faye et al. 2016).

**Figure 1.**
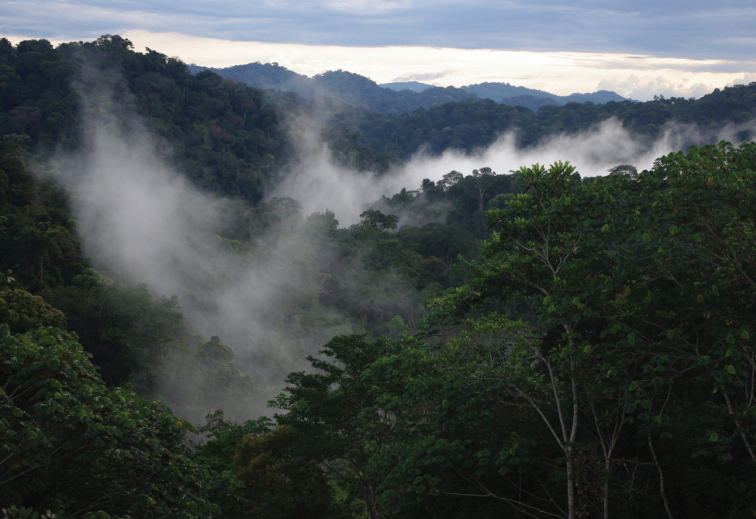
Monts de Cristal National Park, view from Tchimbélé. Photo Thomas L.P. Couvreur

## Results

### Uvariopsis citrata

Taxon classificationPlantaeMagnolialesAnnonaceae

Couvreur & Niangadouma sp. nov.

urn:lsid:ipni.org:names:77156703-1

Figure 2

#### Type.

GABON, Estuaire, Monts de Cristal, near first bridge after Kinguélé village, 0°46'66"N, 10°27'81"E, 14 Jun 2016, *T.L.P. Couvreur 1143* (holotype: WAG!; isotypes: LBV!, P!).

#### Diagnosis.

Resembles *Uvariopsis sessiliflora* (Mildbraed & Diels) Robyns & Ghesquière by the (sub) sessile flowers. Differs from *Uvariopsis sessiliflora* by the strong lemon scent of its crushed leaves or young branches (vs no lemon scent), larger leaves (40 cm vs 10–18 cm), cordate leaf base (vs acute leaf base) and ovoid-conic flowers (vs globose).

#### Description.

Tree 4–7 m tall, 3–5 cm in diameter at breast height (d.b.h.), slash light cream with a black ring, old branches grey, glabrous, young branches light green, pubescent. Leaves distichous, simple, entire, pinnately veined. Petiole 4–5 mm long, 4 mm in diameter, pubescent with short appressed hairs when young, grooved on top, leaf lamina inserted on top. Lamina 45–50 cm long, ca. 12 cm wide, length:width ratio 3.5–4.1, narrowly elliptic to elliptic to narrowly ovate, apex long acuminate, acumen 2–3 cm long, base cordate, coriaceous, glabrous above, glabrous below, strong scent of lemon in crushed leaves; mid rib sunken above, glabrous above, sparsely pubescent below, secondary veins 17–19 pairs, arching 4–5 mm from the margins, tertiary venation network like, raised above and under. Inflorescences cauliflorous (no ramiflorous flowers seen), sparsely spaced along the trunk, more towards the lower half of the trunk, with one to three flowers. Flowers actinomorphic, monoecious, with 6 tepals in total, differentiated in one whorl of 2 sepals and 1 whorl of 4 petals. Male and female flowers similar in size, ovoid-conic, preanthetic flowers seen only. Flowering pedicels male or female 1–2 mm when present, densely covered with short appressed hairs, light brown, up to three bracts tightly packed, covered with short appressed hairs. Sepals male or female 1.3–1.5 cm long, 4 mm wide, length:width ratio 3.5, narrowly ovate, fused for 1/5 to 1/3 of their length, valvate, apex acute, outside densely pubescent with hairs appressed, brown, inside densely pubescent with curly hairs, glabrous towards base. Petals male or female 1.3–1.5 cm long, 7–8 mm wide, 2 mm thick, length:width ratio 3.5, narrowly ovate, apex acute, base truncate, outside densely pubescent with appressed hairs, inside glabrous. In staminate flowers, receptacle conical, 7 mm long, 5 mm wide, stamens numerous, immature, 0.5 mm long, connective truncate, pale yellow. In carpellate flowers, carpels ca. 60, 4–5 mm long, ca. 0.5 mm wide, densely pubescent with long appressed hairs, ovules not observed (preanthetic), stigma cylindrical coiled, glabrous. Fruits unknown.

**Figure 2.**
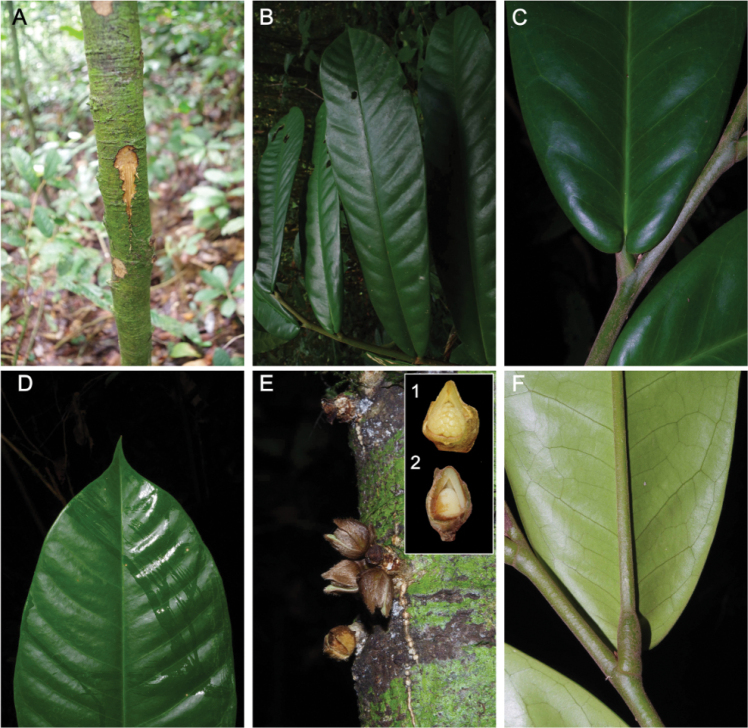
*Uvariopsis citrata*. **A** Trunk with slash (Couvreur 1126) **B** Entire leaf **C** Leaf base upper side **D** Leaf apex, upper side **E** Pre anthetic flower buds on trunk, 1: female flower, 2: male flower **F** Leaf base, lower side. (Couvreur 1143). Photos Thomas L.P. Couvreur.

#### Preliminary conservation status.


 Data deficient. DD. *Uvariopsis citrata* is only known by two collections and three individuals collected in the same area. The type locality is located in a mature forest within the National Park Monts de Cristal, Mbé sector, close to the road that links Kinguélé and Tchimbélé villages. However, the locality does not seem under threat to date and no changes in habitat have been seen in the last few years. However, because it is close to the road and Kinguélé the future of these populations are not certain.

#### Distribution.

Only known to date from Gabon, from one locality in the Monts de Cristal National Park, Mbé sector. 200–300 m in altitude.

#### Habitat.

This species grows in mature or old secondary forests near rivers in periodically inundated soils, or on slopes near rivers.

#### Etymology.

Named after the strong lemon scent of the crushed leaves and young branches one of the diagnostic characters for this species.

#### Paratypes.

**GABON, Estuaire**: Monts de Cristal National Park, Mbé Sector, near first bridge after Kinguélé village, 0°46'66"N, 10°27'81"E, 14 Jun 2016, T.L.P. Couvreur 1126 (WAG!, LBV!, P!)

#### Discussion.

*Uvariopsis citrata* is unique in the genus by the strong lemon smell of its leaves, young branches and young flowers when crushed. This character is rare in African Annonaceae only reported in one other species, also endemic to Gabon, Uvariodendron molundense (Diels) R.E.Fries var. citrata Le Thomas (Le Thomas 1969). This latter species grows in the region of Belinga. To date it remains unclear why some species have this character. Other plants around the type locality did not present this same scent. In addition, *Uvariopsis citrata* has sessile flowers, a character it shares with *Uvariopsis sessiliflora* (Kenfack et al. 2003) endemic to Cameroon. All other *Uvariopsis* species have pedicilate flowers. However, *Uvariopsis sessiliflora* has smaller leaves (10-15 cm) and globose flower buds, while *Uvariopsis citrata* has leaves to 50 cm and ovoid-conic ones. The total number of species in *Uvariopsis* is now 19.

### Laccosperma cristalensis

Taxon classificationPlantaeArecalesArecaceae

Couvreur & Niangadouma sp. nov.

urn:lsid:ipni.org:names:77156704-1

Figure 3

#### Type.

GABON, Estuaire: Monts de Cristal National Park, Mbé sector, forest around the antenna in Tchimbélé, ca. 40 km from Kinguélé, 0°36'21.240"N; 10°24'42.480"E, 13 Jun 2016, *T.L.P. Couvreur 1142* (holotype: WAG!; isotypes: LBV!, P!, G!, K!).

#### Diagnosis.

Resembles *Laccosperma korupensis* Sunderland by the lack of acanthophylls on the cirrus. Differs from *Laccosperma korupensis* by the fewer pinnae (5-8 vs 10-18) sigmoid in shape (vs lanceolate) lacking spines along the margin (vs with spines) and the short 3-5 mm truncated ocrea (vs 8 cm long and tapering to a point).

#### Description.

Clustered, slender palm climbing to 15 m. Stem, circular to oval in cross section, 5-8 mm in diameter; internodes 10–20 cm long. Leaf sheath finely striate, sparsely to moderately armed with fine, green, black tipped, downward and upward pointing spines; sheaths near leaf junction occasionally unarmed; ocrea 3–5 mm long, truncate, round, green, armed with very fine black–tipped spines. Leaves up to 80 cm long; petiole to 3–4 cm long × 4 mm wide, abaxially rounded, adaxially grooved, armed abaxially with short 2–4 mm long, inequidistant, reflexed black spines; rachis to 30 cm long, angular in cross section, armed as the petiole; cirrus to 45 cm long armed as the rachis, although spines become smaller distally; pinnae, 5–8 on each side of the rachis, inequidistant, usually sub-equidistant proximally and borne in pairs distally, sigmoid, finely acuminate at apex, bluntly cuneate at base, 15–16 cm long, 3–4 cm broad at widest point, prominent transverse veinlets, margins lacking spines; acanthophylls absent. Flowers and fruits unknown.

**Figure 3.**
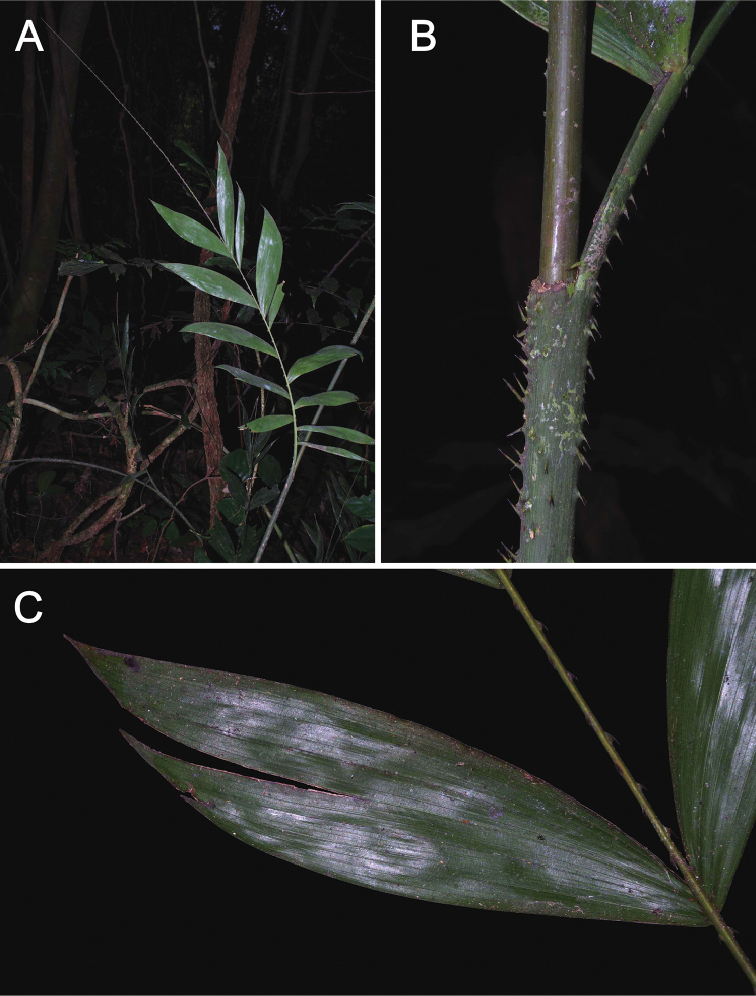
*Laccosperma cristalensis*. **A** Leaf, notice lack of acanthophylls on cirrus **B** Detail of leaf sheath and ocrea **C** Detail of sigmoid pinnae. (Couvreur 1142). Photos Thomas L.P. Couvreur.

#### Distribution.

Only known to date from Gabon, from one locality in the Monts de Cristal National Park, Mbé sector, Tchimbélé.

#### Habitat.

This species was found growing in an old secondary forest, on a slope.

#### Preliminary conservation status.

Data deficient. DD. *Laccosperma cristalensis* is only known by a single collection, thus it is hard to provide an accurate status here. By providing a name to this species, we hope that others will be able to identify potential past collections. The type locality is located in an old secondary forest within the National Park Monts de Cristal, Mbé sector, near Tchimbélé. This forest is, however, close to the telephone antenna area which is regularly maintained by cutting. Important hunting activity was also seen there, suggesting important human activity.

#### Etymology.

Named after the Monts de Cristal National Park in northwestern Gabon, home to an important number of plant species and endemics.

#### Discussion.

*Laccosperma cristalensis* closely resembles *Laccosperma korupensis* by the absence (or near absence) of acanthophylls on the cirrus (extension of the leaf rachis). These are the only two species of African rattans (subtribe Ancistrophyllinae) to share this character. However, *Laccosperma cristalensis* is easily distinguished from *Laccosperma korupensis* by having fewer pinnae (5–8 versus 10–18) which are sigmoid versus lanceolate, lacking spines along the margin versus presence of spines along the margin and an ocrea that is 3–5 mm long and truncated versus 7–10 cm long and tapering to a point (Sunderland 2012). To date none of these species have been collected flowering or fruiting, however, the vegetative characters alone suffice to clearly distinguish them. The total number of species in *Laccosperma* is now 7, although new species are yet to be described from West Africa (Faye et al. 2016).

## Supplementary Material

XML Treatment for Uvariopsis citrata

XML Treatment for Laccosperma cristalensis
